# The impact of multidimensional intergenerational support on fertility intentions among young Chinese couples: the mediating role of parenting burden and fertility anxiety

**DOI:** 10.3389/fpsyg.2025.1734061

**Published:** 2026-01-06

**Authors:** Song Diandian

**Affiliations:** School of Humanities, Southeast University, Nanjing, China

**Keywords:** multi-dimensional intergenerational support, young couples, fertility intentions, childcare burdens, fertility anxiety

## Abstract

**Objective:**

This study examines the impact of multidimensional intergenerational support on fertility intentions among young Chinese couples, alongside the chained mediating mechanisms of childcare burdens and fertility anxiety. Methods: A survey was conducted among 3,246 young married couples (aged 20–35) across 12 Chinese provinces, utilizing the Multidimensional Intergenerational Support Scale, Childcare Burden Scale, Fertility Anxiety Scale, and Fertility Intention Questionnaire to measure multidimensional intergenerational support, childcare burden, parenting anxiety, and fertility intentions.

**Results:**

(1) Multidimensional intergenerational support positively correlated with fertility intentions among Chinese young couples, while negatively correlating with childcare burden and fertility anxiety. Childcare burden positively correlated with fertility anxiety, and both childcare burden and fertility anxiety negatively correlated with fertility intentions. (2) Multidimensional intergenerational support significantly and positively predicted fertility intentions among Chinese young couples. (3) Childcare burden and fertility anxiety, respectively, mediated the effect of multidimensional intergenerational support on fertility intentions among young Chinese couples; (4) Childcare burden and fertility anxiety played a chain-mediated role in the influence of multidimensional intergenerational support on fertility intentions among young Chinese couples.

**Conclusion:**

Multidimensional intergenerational support can directly influence young Chinese couples’ fertility intentions, and can also indirectly influence their fertility intentions through the chained mediating pathway of childcare burden and fertility anxiety.

## 1 Introduction

The persistent decline in fertility rates worldwide has become a shared challenge for many nations. As the world’s most populous nation, China’s declining fertility trend warrants particular attention. Over the past decade, accelerated population ageing has coincided with persistent low birth rates (Gao, 2025), alongside diminished reproductive motivation among younger generations compared to their parents (Luo et al., 2025). This has resulted in China’s total fertility rate remaining persistently at a “very low fertility” level (Lin and Xie, 2019). To address the predicament of low fertility intentions, in 2024 the General Office of the State Council issued the “Several Measures on Accelerating the Improvement of the Fertility Support Policy System and Promoting the Construction of a Birth-Friendly Society” (Government of the People's Republic of China, 2024). This accelerated refinement of the fertility support policy system resulted in a population growth rate of 6.77‰ that year, marking the first increase since 2017 (National Bureau of Statistics, 2025). However, excessive reproductive costs stemming from economic and time pressures continue to impose severe childcare burdens, remaining key negative factors affecting the reproductive intentions of families of childbearing age (Zhou et al., 2025). Moreover, reproductive anxiety has progressively emerged as a significant psychological factor influencing reproductive intentions (Wijma et al., 2002). Notably, intergenerational support—as a vital resource within families—has progressively emerged as a key force alleviating pressures on young couples. This support is multidimensional, encompassing financial, temporal, and emotional dimensions. Grandparents provide comprehensive assistance through economic subsidies (e.g., childcare costs, educational funding), direct childcare involvement (e.g., child supervision, household tasks), and emotional support (e.g., sharing parenting experiences, psychological counselling) (Fu et al., 2023). Young couples aged 20–35 represent a pivotal demographic for reversing declining fertility trends. Exploring the key factors influencing fertility intentions and their underlying mechanisms has thus become a focal point for policymakers and researchers alike. Consequently, surveying their reproductive preferences is crucial for forecasting China’s future demographic trajectory and enhancing fertility rates.

The new family ethos is characterized by a shift in focus, the fusion of parent and child, an emphasis on intimacy, the primacy of the family of origin, individual happiness, family success, and kinship reconfiguration. It is underpinned by the redistribution of intergenerational resources, which is directly linked to the enhanced status of women within the family, particularly that of daughters (Yunxiang, 2024).

From the perspective of new familism, resource exchanges between different generations based on familial blood ties exhibit novel patterns of intergenerational negotiation and cooperation. These encompass emotional support such as emotional comfort and companionship; instrumental support such as caring for grandchildren and assisting with household chores; and financial support such as childbirth subsidies and educational funding. Research indicates that increasing the number of children can enhance elderly care support (Oliveira, 2016). Concurrently, intergenerational financial support, instrumental support, and geographical distance exhibit distinct patterns of influence on fertility. Financial support and proximity to grandparents prove particularly beneficial for urban families’ fertility. Instrumental support appears more advantageous in societies undergoing the second phase of the gender revolution (South Korea and Taiwan) than in societies with stronger gender role constraints (Japan) (Tan, 2023). Furthermore, research indicates that adult daughters’ fertility decisions are highly sensitive to expectations of parental support in time or money (Pessin et al., 2021). Increased emotional closeness between fathers and daughters correlates with heightened willingness among adult daughters to have their first child (Tanskanen and Danielsbacka, 2021). Based on these findings, we propose that multidimensional intergenerational support positively correlates with young couples’ fertility intentions (H1).

### 1.1 The mediating role of childcare burden

The combined pressures families endure in raising children—encompassing financial, temporal, physical and opportunity costs—constitute a comprehensive childcare burden. This burden significantly influences individual fertility intentions and household quality of life, emerging as a highly significant and complex social issue in contemporary Chinese society. According to the 2024 China Child-Rearing Costs Report published (China National Radio Network, n.d.) by the Yuwah Population Research Institute, the average cost of raising a child aged 0–17 nationwide is ¥538,000, while the average cost from birth to undergraduate graduation is approximately ¥680,000. Women raising children aged 0–4 experience a cumulative reduction of 2,106 h in paid work time per week. Each additional child results in a 12–17% reduction in women’s wage rates. This reflects the particularly heavy economic burden Chinese families face in raising children. Economic pressures, scarcity of childcare resources, and psychological anxiety are regarded as key factors constraining fertility intentions (Zhai and Li, 2023). Research indicates independent positive correlations exist between post-pandemic childcare burdens among working women and factors such as working hours, irregular schedules, and time pressure from workload demands (Jeong et al., 2024). Parenting stress and parenting competence can exert both independent and chained mediating effects between family socioeconomic status and paternal involvement in childcare (Song and Wang, 2025). However, other research suggests grandparents are a common source of social support, particularly where financial and instrumental support for childcare can directly alleviate practical burdens on young couples (Sun and Mulvaney, 2021). Wu et al. (2023) contends that balancing intergenerational support, employing digital alternatives, and implementing supplementary social care strategies can mitigate the dual pressures threatening the wellbeing of the sandwich generation serving as family caregivers. Consequently, it is proposed that parenting burden mediates the relationship between multidimensional intergenerational support and young couples’ fertility intentions (H2): higher multidimensional intergenerational support reduces parenting burden, thereby enhancing young couples’ fertility intentions.

### 1.2 The mediating role of fertility anxiety

Reproductive anxiety refers to the worries and unease experienced by individuals of childbearing age throughout the entire process of reproduction, encompassing concerns about their children’s development, the childbirth process, potential negative impacts, their own parenting abilities, and family support (Liangshuang et al., 2025). The root of reproductive anxiety lies in uncertainty about the future and multiple pressures. Individual attributes, family background, sociocultural environment, and policy systems are interrelated, collectively shaping women’s reproductive anxiety (He et al., 2025). Research by Zhao et al. (2024) indicates that perceived stress significantly negatively impacts women’s reproductive intentions. Further studies reveal that when assessing future fertility among female medical students, 58.9% reported experiencing at least some anxiety related to future childbearing (Smith et al., 2023). However, fully recognizing the vital role of intergenerational family support in enhancing reproductive willingness among childbearing-age populations, while leveraging the positive moderating effects of the internet, can help alleviate concerns about “bearing” and “raising” children. Emotional intergenerational support can ease young couples’ anxieties about reproduction, such as worries over pregnancy risks and career impacts, while financial support can reduce anxieties about childcare costs (Liu and Wang, 2023). Based on this, we propose that fertility anxiety mediates the relationship between multidimensional generational support and young couples’ fertility intentions (H3). Specifically, higher levels of multidimensional generational support correlate with lower fertility anxiety, thereby promoting young couples’ willingness to have children.

### 1.3 Chain mediation of childcare burden and fertility anxiety

Each stage of family development—such as the newlywed period, child-rearing period, and empty nest period—presents specific needs and challenges. The child-rearing period is a critical phase for young couples, during which they have heightened demands for parenting resources and financial support. Intergenerational support can bridge gaps in family resources, helping couples adapt to the transition into parenthood. Both parenting burdens and fertility anxiety may serve as mediating variables between multidimensional intergenerational support and fertility intentions. Research indicates a positive dose–response relationship between parenting anxiety and parenting burden, which negatively correlates with maternal quality of life (QOL) (Mishina et al., 2012). Higher parenting burdens may further trigger fertility anxiety—for instance, couples devoting substantial time to childcare may worry about work-family imbalance, leading to anxiety about having additional children. Based on this, we propose that childcare burden and fertility anxiety play a chain-mediated role in the relationship between multidimensional intergenerational support and young couples’ fertility intentions (H4).

**Figure 1:**
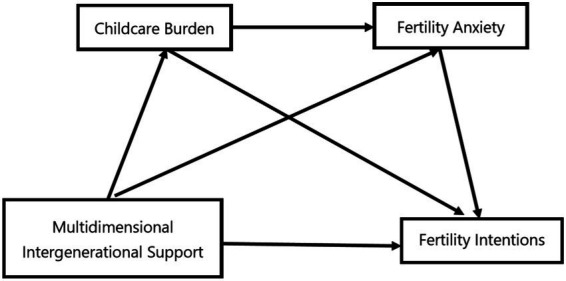
Theoretical framework of the serial mediation model.

In summary, multidimensional intergenerational support is crucial for promoting fertility, yet empirical evidence remains limited regarding the mediating roles of childcare burdens and fertility anxiety. Therefore, this study primarily focuses on the group of Chinese couples of childbearing age, with three main objectives: (1) In the field of demography, it enriches research on the micro-mechanisms shaping fertility intentions by linking intergenerational support to psychological factors such as fertility anxiety and practical factors like childcare burdens; (2) Within the field of family sociology, the application of neo-familialism theory has been extended to the context of low fertility, highlighting the multidimensional characteristics of intergenerational support; (3) Within the field of psychology, this study has revealed the chain-like mediating role of negative psychological states such as anxiety and real-world burdens like childcare in the relationship between intergenerational support and behavioral willingness. The conceptual model is illustrated in Figure 1.

## 2 Materials and methods

### 2.1 Participants

This study employed stratified random sampling across 12 provinces in China (including Beijing, Shanghai, Guangdong, Sichuan, Henan, etc.) to ensure regional representativeness. Inclusion criteria were: (1) married couples aged 20–35; (2) no history of severe physical or mental illness; (3) voluntary participation with written informed consent.

Data collection occurred from December 2024 to February 2025, combining an online questionnaire platform (Wenshuangxing) with in-person interviews. A total of 3,500 questionnaires were distributed, with 3,246 valid responses collected, yielding a valid response rate of 92.74%. This study strictly adhered to the ethical principles outlined in the 1964 Declaration of Helsinki and its subsequent amendments.

### 2.2 Measurement tools

#### 2.2.1 Multidimensional intergenerational support scale

Based on the intergenerational support scale developed by the Population Research Institute at Xi’an Jiaotong University, which measures children’s provision of financial support, daily care, and emotional comfort to their parents (Zhang and Li, 2005), a “Intergenerational Support Scale” was developed to measure parental provision of financial support, daily care, and emotional comfort to children. It comprises three dimensions—emotional support, instrumental support, and financial support—with 15 items rated on a 5-point Likert scale (1 = ‘Never’ to 5 = “Always”). In this study, Cronbach’s alpha coefficients for the total scale and its three dimensions were 0.92, 0.88, 0.86, and 0.84, respectively.

#### 2.2.2 Parental burden assessment scale

The Parental Burden Scale was developed based on the Parenting Stress Inventory Short Form (PSI-SF) (Luo et al., 2019). It comprises 8 items rated on a 5-point Likert scale (1 = “Strongly disagree” to 5 = “Strongly agree”), with higher scores indicating greater parental burden. In this study, the Cronbach’s alpha coefficient for this scale was 0.87.

#### 2.2.3 Fertility anxiety scale

The Fertility Anxiety Scale, developed based on the Fertility Anxiety Scale for Women of Childbearing Age (Chen et al., 2025), comprises 10 items. It measures two dimensions: practical anxiety and psychological anxiety. Scores are rated on a 5-point Likert scale (1 = “Strongly disagree” to 5 = “Strongly agree”), with higher scores indicating greater fertility anxiety. In this study, the Cronbach’s alpha coefficient for this scale was 0.89.

#### 2.2.4 Fertility desire questionnaire

The Fertility Desire Questionnaire was designed based on the Psychometric Properties of Fertility Desire Scale (Naghibi et al., 2019). It employs a 5-point Likert scale (1 = “Strongly Disagree” to 5 = “Strongly Agree”), with higher scores indicating greater fertility anxiety. In this study, the Cronbach’s alpha coefficient for this scale was 0.83.

### 2.3 Data analysis methods

Data analysis was conducted using SPSS 26.0 and Mplus 8.3. Descriptive statistics and correlation analysis were performed in SPSS 26.0 to examine relationships among variables. A chained mediation model was constructed in Mplus 8.3, employing Bootstrap sampling (1,000 iterations) to test the mediating effects of parenting burden and fertility anxiety on the relationship between multidimensional intergenerational support and fertility intentions among young Chinese couples.

## 3 Results

### 3.1 Common method bias test

Using Harman’s single-factor method, an unrotated exploratory factor analysis (EFA) was conducted on all measurement items of multidimensional intergenerational support, childcare burden, fertility anxiety, and fertility intention. Five factors with eigenvalues greater than 1 were obtained. The first common factor extracted explained 28.73% of the variance, which is less than 40%. This indicates no severe common method bias exists.

### 3.2 Descriptive statistics and correlation analysis

Descriptive statistics for key variables are presented in Table 1. (1) Multidimensional intergenerational support showed a significant positive correlation with fertility intention (*r* = 0.42, *p* < 0.001); (2) Multidimensional intergenerational support showed significant negative correlations with childcare burden (*r* = −0.38, *p* < 0.001) and fertility anxiety (*r* = −0.35, *p* < 0.001); (3) Childcare burden was significantly positively correlated with fertility anxiety (*r* = 0.51, *p* < 0.001); (4) Both childcare burden (*r* = −0.39, *p* < 0.001) and reproductive anxiety (*r* = −0.41, *p* < 0.001) showed significant negative correlations with reproductive intentions. These findings provide preliminary evidence supporting the research hypotheses.

**Table 1:** Descriptive statistics and correlation analysis of variables.

Variable	Mean ± SD	1	2	3	4
1. Multidimensional intergenerational support	3.42 ± 0.86	1			
2. Parenting burden	3.15 ± 0.78	−0.38***	1		
3. Fertility anxiety	3.21 ± 0.81	−0.35***	0.51***	1	
4. Fertility intention	2.53 ± 0.72	0.42***	−0.39***	−0.41***	1

**p < 0.01; ***p < 0.001; *N* = 3,246 couples.

### 3.3 Testing the chain mediation effect

This study employed Mplus 8.3 software to construct a chain mediation model. With multidimensional intergenerational support as the independent variable, fertility intention as the dependent variable, and childcare burden and fertility anxiety as mediating variables, the model demonstrated excellent fit. All fit indices met the recommended standards for academic research (see Table 2). χ^2^/df = 2.87 (χ^2^ = 563.21, df = 196), below the critical value of 3, indicating overall good model fit. The Comparative Fit Index (CFI) = 0.95 and Tucker-Lewis Index (TLI) = 0.94, both exceeding 0.90, reflect high structural validity of the model. The Root Mean Square Error of Approximation (RMSEA) = 0.042 (95% confidence interval [0.038, 0.046]), which is below the acceptable threshold of 0.08 and whose confidence interval does not include 0.08, indicates a small prediction error of the model; Standardized Root Mean Square Residual (SRMR) = 0.039, below 0.08, further validating the model’s high fit with the data and its suitability for subsequent mediation effect analysis.

**Table 2:** Decomposition of serial mediation effects.

Effect type	Path description	Effect value	Standard error (SE)	*t*-value	95% Confidence interval	*p*-value	Proportion of total effect (%)
Direct effect	Intergenerational support → Fertility intention	0.26	0.02	6.83	[0.10, 0.18]	<0.001	54.17
Indirect effect 1	Intergenerational support → Parenting burden → Fertility intention	0.08	0.02	5.27	[0.08, 0.15]	<0.001	16.66
Indirect effect 2	Intergenerational support → Fertility anxiety → Fertility intention	0.09	0.02	4.91	[0.07, 0.13]	<0.001	18.75
Indirect effect 3 (Serial)	Intergenerational support → Parenting burden → Fertility anxiety → Fertility intention	0.05	0.01	4.15	[0.05, 0.09]	<0.001	10.42
Total indirect effect	–	0.22	0.02	7.36	[0.24, 0.32]	<0.001	45.83
Total effect	–	0.48	0.03	8.51	[0.37, 0.47]	<0.001	100.00

**Figure 2:**
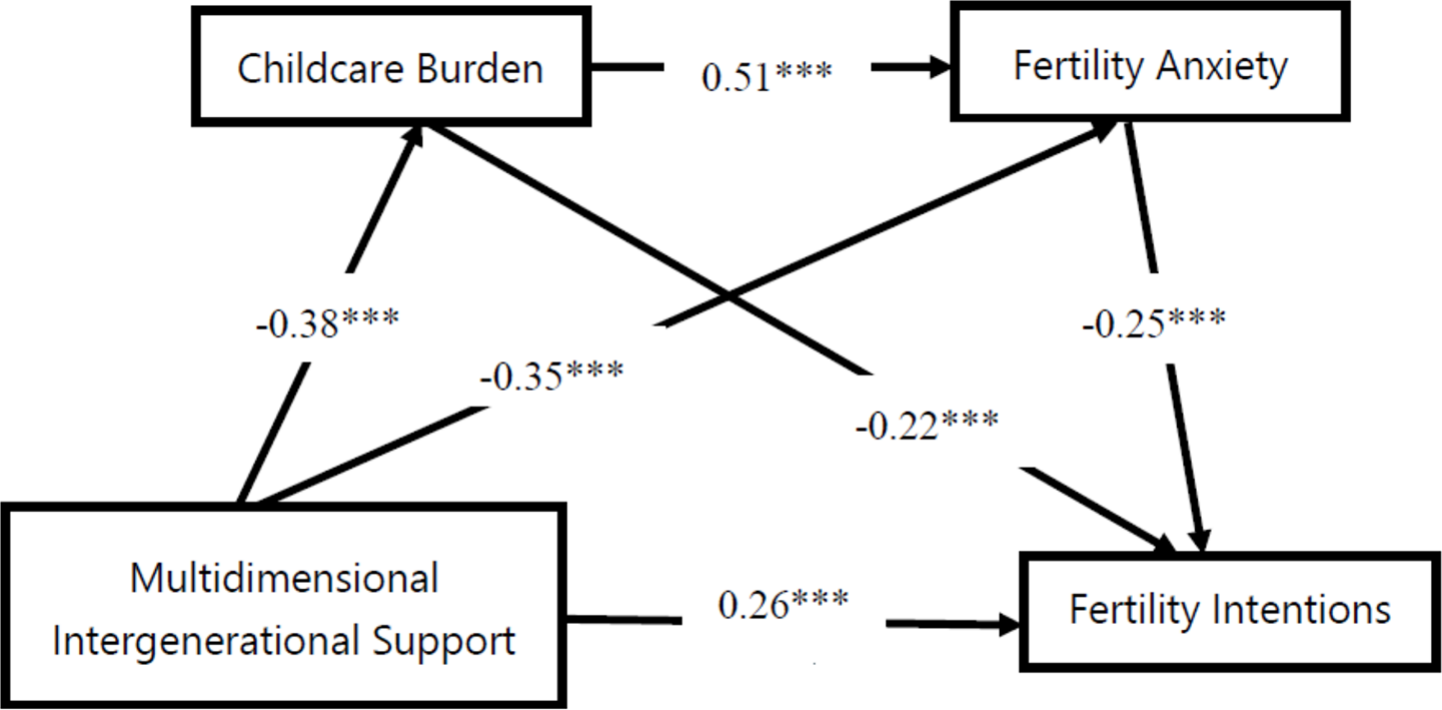
Serial mediation model of parenting burden, fertility anxiety, multidimensional intergenerational support, and fertility intention. ***p < 0.01, **p < 0.05.

First, we examined the direct predictive effect of multidimensional intergenerational support on fertility intentions among young Chinese couples. Results revealed a significant direct effect of multidimensional intergenerational support on fertility intentions (*β* = 0.26, *t* = 6.83, *p* < 0.001), indicating that intergenerational support directly and positively promotes fertility intentions even when controlling for mediating variables. Second, after incorporating mediating variables into the model, we found that multidimensional intergenerational support was negatively correlated with childcare burden (*β* = −0.38, *t* = −12.65, *p* < 0.001), childbearing anxiety (*β* = −0.35, *t* = −11.425, *p* < 0.001), indicating that multidimensional intergenerational support effectively alleviates childcare burdens and fertility anxiety among young Chinese couples. Childcare burden (*β* = −0.22, *t* = −7.54, *p* < 0.001) and fertility anxiety (*β* = −0.25, *t* = −8.21, *p* < 0.001) were negatively correlated with fertility intentions; Parenting burden and reproductive anxiety were positively correlated (*β* = 0.51, *t* = 16.93, *p* < 0.001). The predictive relationships among variables are illustrated in Table 3 and Figure 2.

**Table 3:** Regression analysis results of variable relationships in the mediation model.

Regression equation variable		Overall fit index of the equation	Significance of regression coefficients	
Outcome variable	Predictor variable	(*R*^2^)	(*β*)	(*t*-value)
Parenting burden	Multidimensional intergenerational support	0.14	−0.38	−12.65***
Fertility anxiety	Multidimensional intergenerational support	0.28	−0.35	−11.42***
	Parenting burden		0.51	16.93***
Fertility intention	Multidimensional intergenerational support	0.36	0.26	6.83***
	Parenting burden		−0.22	−7.54***
	Fertility anxiety		−0.25	−8.21***

***p < 0.001, **p < 0.01, *p* < 0.05.

Finally, the mediation effect results were tested using bootstrap bias correction, with 95% confidence intervals calculated through 1,000 repeated samples. The mediation analysis revealed a direct effect value of 0.26 (*p* < 0.001, 95% CI [0.10, 0.18]), accounting for 54.17% of the total effect. The total indirect effect was 0.22 (*p* < 0.001, 95% CI [0.24, 0.32]), accounting for 45.83% of the total effect. Since the confidence intervals did not include zero, both the direct and indirect effects were statistically significant. The mediating effect comprised three pathways: (1) Intergenerational support → Childcare burden → Fertility intention, with an indirect effect value of 0.08 (*p* < 0.001, 95% CI [0.08, 0.15]), accounting for 16.66% of the total effect, indicating a significant indirect effect. (2) Intergenerational support → Fertility anxiety → Fertility intention, with an indirect effect value of 0.09 (*p* < 0.001, 95% CI [0.07, 0.13]), accounting for 18.75% of the total effect, indicating a significant indirect effect. (3) The chain indirect effect from generational support → childcare burden → childbearing anxiety → childbearing intention was 0.05 (*p* < 0.001, 95% CI [0.05, 0.09]), accounting for 10.42% of the total effect, indicating a significant indirect effect. Results are shown in Table 2.

## 4 Discussion

### 4.1 Direct positive impact of multidimensional intergenerational support on fertility intentions

Research findings indicate that multidimensional intergenerational support significantly and positively predicts fertility intentions among young Chinese couples, validating Hypothesis H1. This result aligns with previous studies. Sear (2017) demonstrated that intergenerational assistance (from grandparents to parents and children) is a cross-cultural phenomenon, suggesting that the availability of relatives may be a significant factor in determining whether and when to have children. Intergenerational support exerts a significant overall positive influence on the desire to have a second child (Fu et al., 2023). Additionally, research indicates that downward intergenerational support in living arrangements and financial assistance significantly increases the probability of family reproductive behavior (Yu, 2024). The social intergenerational support theory highlights the economic reciprocity, mutual assistance in daily life, emotional support, and shared life experiences and resources between family generations (Mu, 2002). This support significantly influences the number of children offspring expect to have, as well as the gap between their desired fertility and actual fertility. Moreover, it is transmitted through intergenerational support, continuously reinforcing the fertility intentions of subsequent generations.

### 4.2 Mediating role of childcare burden and fertility anxiety between multidimensional intergenerational support and fertility intentions

This study reveals that beyond direct associations, childcare burden and fertility anxiety significantly mediate the relationship between multidimensional intergenerational support and fertility intentions. First, childcare burdens negatively correlate with young couples’ fertility intentions and mediate the relationship between multidimensional intergenerational support and fertility intentions, consistent with Hypothesis H2. This finding supports research by Chen et al. (2024), indicating that childcare stress significantly suppresses the desire to have more children. The economic theory of fertility (Leibenstein, 1957) posits that couples decide to have children only when the marginal utility of a child exceeds its marginal disutility. Economic conditions act as a constraint, influencing fertility decisions across different population groups, communities, and income levels alongside systemic barriers like costs (Ronnenberg and Conrad, 2024). Qiao et al. (2024) identifies economic pressure and time constraints as primary barriers to childbearing. Additionally, research indicates that reductions in childcare costs, primary education expenses, and secondary education expenditures exert statistically significant effects on fertility decisions (Shin, 2008). Alleviating the financial burden of childrearing and increasing discretionary time for couples of childbearing age are significantly correlated with their willingness to have more children (Zhu et al., 2022). Moreover, intergenerational financial support effectively alleviates childcare burdens. For instance, such support reduces reproductive burdens for women with lower human capital, thereby increasing their fertility intentions (Wang and Yu, 2025). Intergenerational financial assistance significantly influences fertility intentions among individuals aged 31–35 who either lack property ownership or possess two or more properties (Jiang and Zhang, 2025). Based on this, multidimensional intergenerational support alleviates young couples’ pressures in time allocation and financial expenditure by providing practical childcare assistance—such as grandparent childcare, household help, and financial subsidies—thereby reducing the “burden fear” associated with raising multiple children and ultimately boosting fertility intentions.

Second, this study confirms Hypothesis H3: fertility anxiety negatively correlates with young couples’ fertility intentions and mediates the relationship between multidimensional intergenerational support and fertility intentions—consistent with prior research. Anxiety related to pregnancy, childbirth, and parental roles all negatively correlates with fertility intentions (Zhang et al., 2022). Although anti-fertility anxiety is more deeply rooted in micro-level expressions—emphasizing reproductive autonomy and affirmation of motherhood within broader feminist awakening contexts (Li and Zhou, 2025)—young couples experience anxiety when confronted with societal pressures and unequal family burdens. This arises from feelings of insecurity and powerlessness when subjective fertility intentions clash with objective obstacles. Jiang et al. (2022) found that anxiety disorders significantly mediate the relationship between work–family conflict and third-child fertility intentions among working women of childbearing age. In this study, reproductive anxiety, as a negative psychological variable, positively influences fertility intentions by reducing negative emotions. Based on this, multidimensional intergenerational support, as a resource provider, enhances positive psychology and reduces negative psychology. It offers psychological comfort, shares parenting experiences, and provides economic security for young couples. This support reduces their anxieties about reproductive risks, career impacts, and parenting capabilities, thereby strengthening their fertility intentions.

### 4.3 Chain mediation effect of childcare burden and fertility anxiety

This study further reveals that childcare burden and fertility anxiety exert a chain mediation effect on the influence of multidimensional intergenerational support on young Chinese couples’ fertility intentions, validating Hypothesis H4 and aligning with findings in this research field. Empirical findings indicate that among reproductive utility values held by young adults of childbearing age, individual emotional utility exerts the strongest positive predictive effect on fertility plans. Anxiety over indirect costs reduces the probability of fertility plans more significantly than anxiety over explicit costs (Li and Wu, 2022). Specifically, young couples’ reproductive anxiety does not arise in isolation but largely stems from concerns about future childcare burdens. When anticipated childcare burdens are perceived as excessive—such as a lack of childcare support or significant financial pressure—it triggers anxieties about work-family imbalance and inadequate child rearing, thereby reducing fertility intentions. Xu et al. (2023) found that women are more concerned about balancing work and childcare, and that economic conditions influence fertility intentions. Lei and Phakdeephirot (2023) emphasizes that robust supporting measures for childbearing are crucial for enhancing fertility intentions. This study proposes a chain-mediated logic: “Real-world burdens → Negative psychological states → Fertility intentions.” This mechanism reveals a dual pathway through which intergenerational support influences fertility intentions: alleviating real-world burdens lays the groundwork for reducing psychological anxiety. These factors do not operate independently but form a progressive chain of effects. Material burdens serve as precursors to psychological anxiety. Reducing childcare burdens effectively lowers fertility anxiety, while intergenerational support alleviates burdens through tangible assistance, thereby curbing anxiety at its source and ultimately boosting fertility intentions.

## 5 Research limitations

This review also has several limitations. First, existing studies predominantly utilize cross-sectional data, making it difficult to establish causal relationships and dynamic changes between variables. Second, current research primarily focuses on the influence of grandparents—the providers of intergenerational support—on their grandchildren’s fertility intentions, while paying insufficient attention to the grandchildren’s own support needs and expectations. Finally, existing studies predominantly examine traditional forms of intergenerational support such as financial assistance, labor support, and emotional support, while neglecting newer forms emerging in modern society, such as remote support and digital support. Future research should increasingly adopt longitudinal designs to better understand the causal relationships and developmental trajectories among intergenerational support, childcare burdens, fertility anxiety, and fertility intentions. Additionally, future studies should place greater emphasis on the subjective experiences and needs of young couples, as well as emerging forms of intergenerational support. This approach would explore the impact of intergenerational support on fertility intentions from the perspective of matching supply and demand.

## 6 Conclusion

Multidimensional intergenerational support positively predicts fertility intentions among young Chinese couples, with childcare burden and parenting anxiety serving as independent mediators and chain mediators, respectively, in this process. The chain mediation effect further indicates that multidimensional intergenerational support can mitigate the relationship between childcare burden and parenting anxiety and fertility intentions among young Chinese couples. These findings provide crucial insights into the interplay between intergenerational support, childcare burdens, parenting anxiety, and fertility intentions, holding significant implications for developing effective fertility support policies and interventions.

## Data Availability

The raw data supporting the conclusions of this article will be made available by the authors, without undue reservation.
